# Drivers of Live Cattle Price in the Livestock Trading System of Central Cameroon

**DOI:** 10.3389/fvets.2017.00244

**Published:** 2018-01-17

**Authors:** Paolo Motta, Ian G. Handel, Gustaf Rydevik, Saidou M. Hamman, Victor Ngu Ngwa, Vincent N. Tanya, Kenton L. Morgan, Barend M. deC. Bronsvoort, Thibaud Porphyre

**Affiliations:** ^1^Royal (Dick) School of Veterinary Studies, The Roslin Institute, University of Edinburgh, Easter Bush, Midlothian, United Kingdom; ^2^The European Commission for the Control of Foot-and-Mouth Disease (EuFMD), Food and Agriculture Organization of the United Nations, Rome, Italy; ^3^Royal (Dick) School of Veterinary Studies, University of Edinburgh, Midlothian, United Kingdom; ^4^Institute of Agricultural Research for Development, Regional Centre of Wakwa, Ngaoundere, Cameroon; ^5^School of Veterinary Medicine and Sciences, University of Ngaoundere, Ngaoundere, Cameroon; ^6^Cameroon Academy of Sciences, Yaoundé, Cameroon; ^7^School of Veterinary Science, Institute of Ageing and Chronic Disease, University of Liverpool, Neston, Wirral, United Kingdom

**Keywords:** cattle, price formation, Cameroon, markets, trading system

## Abstract

Livestock production and trade are critical for the food security and welfare of rural households in sub-Saharan Africa. In Cameroon, animal trade consists mainly of live cattle commercialized through livestock markets. Identifying the factors contributing to cattle price formation is critical for designing effective policies for sustainable production and for increasing food availability. In this study, we evaluated the influence of a range of individual- and market-level factors on the price of cattle that were sold in all transactions (*n* = 118,017) recorded over a 12-month period from 31 livestock markets in the main cattle production area of the country. An information-theoretic approach using a generalized additive mixed-effect model was implemented to select the best explanatory model as well as evaluate the robustness of the identified drivers and the predictive ability of the model. The age and gender of the cattle traded were consistently found to be important drivers of the price (*p* < 0.01). Also, strong, but complex, relationships were found between cattle prices and both local human and bovine population densities. Finally, the model highlighted a positive association between the number of incoming trading connections of a livestock market and the price of the traded live cattle (*p* < 0.01). Although our analysis did not account for factors informing on specific phenotypic traits nor breed characteristics of cattle traded, nearly 50% of the observed variation in live cattle prices was explained by the final model. Ultimately, our model gives a large scale overview of drivers of cattle price formation in Cameroon and to our knowledge is the first study of this scale in Central Africa. Our findings represent an important milestone in designing efficient and sustainable animal health management programme in Cameroon and ensure livelihood sustainability for rural households.

## 1 Introduction

In Cameroon, the livestock sector contributes 20% of the agricultural gross domestic product (GDP), with trade of live animals and livestock products representing a major component of the agricultural sector (1). As in most of sub-Saharan Africa (SSA), livestock production is particularly important for rural populations, providing year-round employment opportunities and a key source of revenues (2, 3). Notably, the sale of livestock, mainly cattle, is a rapid cash generating mechanism (4) that allows purchasing food and family necessities (3, 5) as well as representing a source of self-insurance against income shocks caused by unforeseen events impacting rural households (5). Livestock, and its associated economic value, represents a key asset for reducing the vulnerability of rural households to a number of external factors, such as climate change, diseases, and social and political instability. However, the strategies available to these households for reducing the severity of animal health and welfare issues on livestock value are generally weak and inadequate (5).

Various studies have been carried out in Cameroon to evaluate the burden of livestock and zoonotic diseases (6–12), identify constraints for disease controls in pastoral and small-scale livestock husbandry and production system (13), and better understand how the cattle trade is structured (14). Together, these studies provide a collection of information which would enable the veterinary services to better design animal health management programmes in Cameroon. However, should these programmes be implemented in the field, their sustainability and efficiency would depend on their degree of integration within the local livestock production system, notably by ensuring a minimum socio-economic impact while guaranteeing benefits for both national and household economies. Developing integrated animal health management programmes, therefore, requires a better understanding of the place (and the perceived value) of livestock in the local economy against which the acceptability and feasibility of intervention would be measured (15).

In this context, a better understanding of the value of livestock represents an important stepping stone in developing efficient and robust, evidence-based animal health interventions. Indeed, knowledge of livestock value represents a key component of any efforts to estimate the economic burdens of infectious diseases (16) and assess the economic benefits of alternative prevention and control strategies (17). However, most economic assessments of animal health strategies assumed livestock farmers sell a homogeneous product with a fixed and constant value. While this assumption may not affect estimated net gain of tested strategies in settings where the price variability is limited, problems may arise in situations where variations in trade behavior vary in time and/or space. In particular, trade behavior may vary in response to environmental and ecological factors, such as droughts and pasture availability, and to shifts in export and meat demand (18), including those related to religious festivities and national celebrations (19). These variations could directly affect the value of livestock and, as a consequence, increase (potentially drastically) the complexity in the socio-economic impact of veterinary intervention strategies on national and household economies and ultimately undermine the success of implemented animal health programmes.

The lack of a reliable price information system is a common constraint when attempting to improve our understanding of livestock marketing systems in both the pastoral production and urban consumption areas across most of SSA (20, 21). In Cameroon, livestock trade is predominantly represented by sales of cattle in conventional trading infrastructures (1, 14) regulated by the Municipalities and by the local Delegations of the Ministry of Livestock, Fisheries and Animal Industries (MINEPIA). In markets located in the Adamawa and West Regions of Cameroon, two of the main cattle production areas of the country, details on prices, provenance and characteristics of each animal traded are recorded (in various forms) and kept locally mainly for tax collection purposes. Once collected and centralized in a unique dataset, these records represent a unique resource for better understanding the factors that drive formation of live cattle price in Cameroonian trade system. In the current study, the aim was to use this collection of market records to evaluate which animal and market factors contribute to cattle price formation and estimate how these factors influence live cattle prices within the livestock trading system of the major livestock production areas of Cameroon. Specifically, we developed a generalized additive mixed-effect model and applied an information-theoretic approach derived from the ecology literature (22) to identify factors (as well as evaluating their robustness) impacting on the value of traded cattle in Cameroonian markets.

## 2 Materials and Methods

### 2.1 Study Population

In this study, all markets that are involved in the trade of cattle and are located in the West, North-West, and Adamawa Regions of Cameroon were considered for inclusion. These include all markets listed in the livestock market registers from the relevant Regional Delegation of the MINEPIA of the West, North-West, and Adamawa Regions of Cameroon (*n* = 52) as well as additional markets (*n* = 7) identified through interviews with veterinary officials and market managers (14).

From the 59 markets considered in this study, all data related to cattle transactions reported in official market records were obtained for a 12-month period from September 2013 to August 2014. In Cameroon, details of cattle transactions in markets are recorded on paper and handwritten. Market records were therefore scanned using a portable wireless scanner and a smartphone, manually transcribed to an electronic database by two persons separately and cross-checked for discrepancies. When erroneous or missing records were identified, original scans were re-examined and data re-entered in the database. Semi-structured interviews were also conducted with the veterinary officials and market managers using French and English (Cameroonian official languages) to gather additional background information regarding the transaction process and the roles of the different stakeholders involved in the negotiations.

**Figure 1:**
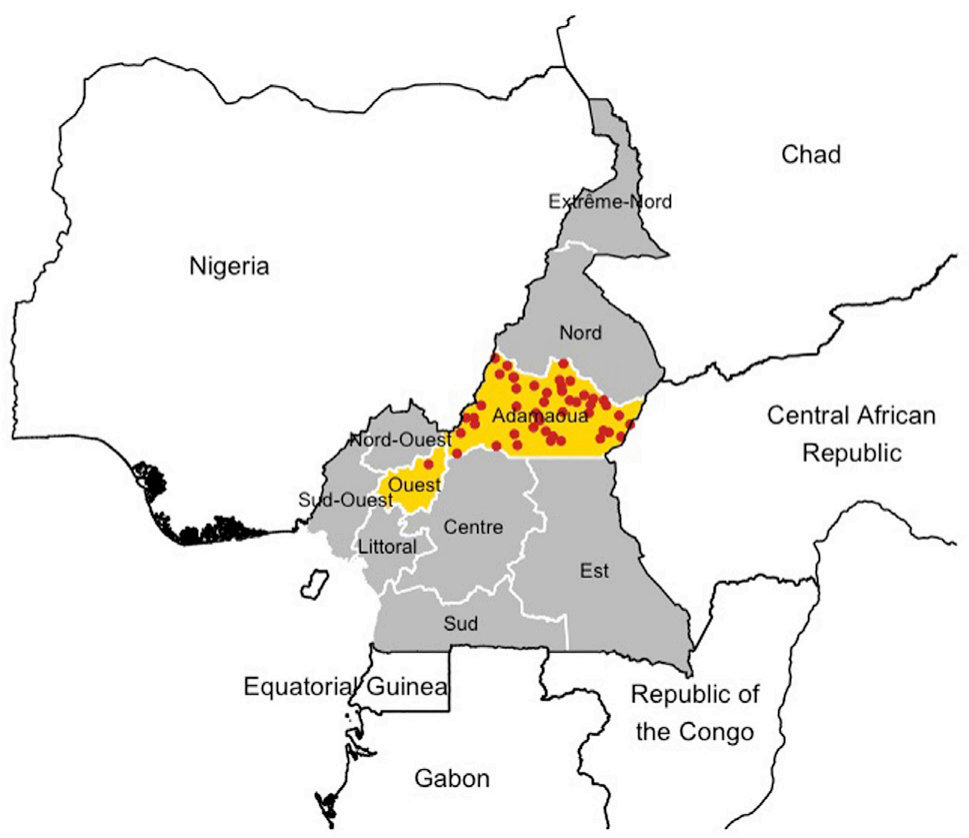
Study area and locations of the livestock markets included in the analysis. The Regions in yellow highlight the areas where data collection was carried out and the red dots refer to the locations of the markets.

Among all markets considered in this study, only 31 cattle markets (53%) recorded detailed information for each individual transaction occurring within the study period. In total, 118,017 cattle transaction records were extracted from the archives of these 31 markets. Figure 1 shows the study area as well as the location of the 31 markets involved in the current study.

Briefly, the study area covers the Adamawa and The West (“Ouest”) Region of Cameroon (Figure 1). The Adamawa Region is mainly a pastoral highland above 1,000 m, of approximately 64,000 km^2^ and considered to be the main livestock production area of Cameroon with an official cattle population of about 1,250,000 head of cattle (23) and an open woodland Guinea savannah ecotype. It is both a source and a destination of transhumant herds originating from other areas of the country during the dry season (October to April). The West Region is a lower lying area of 14,000 km^2^ with a much smaller cattle population of about 160,000 heads (23) and a more Sahel woodland savannah ecotype. Together, both Regions contain about a quarter of the national cattle herd.

### 2.2 Predictor Variables

The extracted 118,017 records of cattle transaction considered in this study included not only the price (in Central African Franc, CFA) at which individual animals have been traded but also the date (in week) and location (market name and administrative Division) of the transaction. To account for potential seasonal variations in the price of live cattle, transactions were determined to have occurred during either the dry or the rainy season. For the purpose of this study, the dry season was considered to be occurring between October and April, and the rainy season between May and September. For each transaction, details of several individual-level characteristics of the traded animal were also reported, including the village or the market of provenance of the animal, its age and its sex. All cattle traded were also recorded as belonging to one of the following specific commodity types: “steer,” “bull,” “cow,” “young bull,” or “heifer.”

Over the entire dataset, information on price, commodity types, date, and location were complete for each recorded transaction. However, nearly 20% of the official records showed missing information on the age of the animal traded. To avoid loss of information, missing information on cattle age was imputed using a matching imputation approach (24). Briefly, we assumed that missing information was missing completely at random and represented a random subset of the data. As such, age information was imputed by matching known details of the animals for which age is missing with those of animals for which age was reported. Here, we matched animals based on their specific commodity type (i.e., steer, bull, cow, young bull, or heifer), the market at which they were sold and the month of the transaction. Figure S1 in Supplementary Material compares the age distribution from the incomplete dataset with that from the dataset including the imputed observations. Although the general age distribution remains consistent, there were some clear errors when inferring the exact individual age. Particularly, 3- and 4-year old animals were difficult to discriminate, while age of >10 years old animals were difficult to estimate with certainty. To avoid biases, cattle were therefore regrouped into five age categories levels with similar age intervals: ≤2 years old, between 3 and 4 years old, between 5 and 6 years old, between 7 and 10 years old, and >10 years old.

Details of markets in which transactions occurred were also included in the data. The total number of transactions that occurred in each market was used as a proxy for their size and, thus, their importance in the trade system in Cameroon. However, the precision of such a measure is limited as it does not consider the global structure of the trade system and disregard markets potential to access others. To capture this feature, we considered cattle markets in Cameroon to form a large network, where markets are “nodes” of the network that are directly linked by the movement of cattle that were purchased and/or sold. The formal analysis of the cattle trade network in Cameroon has been previously published in Ref. (14), including the study of several network centrality measures (including degree, in- and out-degree, betweenness, and eigenvector) computed to extract the position of markets in the trade network and identify which markets are more “central” than others. Detailed definitions of the considered centrality measures and their characteristics are provided in Ref. (14). However, in the present analysis, only in-degree was considered as an indicator of network position for markets. This is because (i) centrality measures are highly correlated (25), particularly in regard to the cattle trade network in Cameroon (Table S1 in Supplementary Material); (ii) the estimate of in-degree can be easily and reliably calculated from transaction records in Cameroon; and (iii) the estimate of in-degree is relative stable, even in situation where incomplete network information occurs (26).

We further recorded the geographical location of each cattle market using a Global Positioning System (GPS) at the time of the data collection. From the obtained latitude and longitude of markets, estimates of human and cattle population densities were extracted from raster datasets freely available online (27, 28) to account for the distribution of populations and their potential impact on the local demand and supply capacity for cattle meat. We assumed that the individual demand for cattle meat is directly related to local human density, whereas supply would be affected by the local density of cattle. For the purpose of analysis, we further assumed that both cattle and human populations were stable overtime and could be extrapolated from historical, though recent, information. Data on cattle population density were extracted for the year 2005 from the Gridded Livestock of the World (GLW) version 2.0 (29), freely available at the FAO GeoNetwork repository (27). Information on the human population density the year 2010 was obtained from the WORLDPOP data (28).

### 2.3 Statistical Analysis

#### 2.3.1 Modeling Framework

A generalized additive mixed-effect modeling approach (GAMM) (30) was used to estimate the effect of animal- and market-level factors influencing the value of cattle in the study area during the period September 2013 to August 2014. For this analysis, the outcome was the price (in thousand CFA), *Y_ij_*, of animal *i* at market *j*. Here, the price *Y_ij_* was assumed to be linearly dependent on a set of *k* predictor variables *x_k_* and on *m* unknown smoothing functions *f_m_* of non-linear predictor variables *z_m_* such that:

(1) $$g(E(Y_{ij}))=\alpha +\frac{\Sigma }{k}\beta_{k}x_{ki}+\frac{\Sigma }{m}f_{m}(z_{mi})+U_{j}+\epsilon_{ij}$$

where *g*(*E*(*Y_ij_*)) is the linear link function of the expectation of the price *E*(*Y_ij_*); *α* is the intercept; *β_k_* are the coefficients of the assumed independent predictors *x_k_*; and $\epsilon_{ij}$ is the error term or “residuals.” A random effect term *U_j_* was added to account for correlation arising from repeated information from the same market.

Animal and market-level characteristics of each transaction were considered as predictor variables in the model. To comply with the underlying assumption of independence, relationship between predictor variables was visually screened, and their correlation was evaluated by computing the Pearson’s correlation coefficient *r*. Variables showing *r* > 0.6 were identified and only the biologically or economically most relevant variables were considered for the modeling. In particular, market size, as defined by the number of traded cattle over a 12-month period, was highly correlated with in-degree centrality (*r* = 0.62) and was therefore discarded from further analysis.

Finally, preliminary analyses highlighted the non-linear relationship of the cattle price with both the local bovine population density and the week of the transaction (see Figures S2 and S3 in Supplementary Material). As such, both variables were included in our model as non-linear predictor variables *z_m_* and were modeled using penalized regression splines. Details for the nine independent variables that were considered as potential drivers of cattle value in markets and use in the analysis are given in Table 1.

**Table 1:** Variables used to build the modeling approach of cattle price at the market level.

Name	Variable	Data type	Definition
AGE	Age	Ordinal	A categorized variable with five levels (<2, 3–4, 5–6, 7–10, >10 years old)
SEX	Sex	Binary	Male or female
SEAS	Season	Binary	Season at which transaction occurred: dry (October to April) or rainy (May to September)
DIV	Division	Categorical	Names of the six Divisions: Djerem, Faro et Deo, Mayo Banyo, Mbere, Vina and Noun)
CD	Cattle density	Continuous	Density of cattle living in the administrative area of the market as extracted by the online repository varied between 0 and 30 animals per km^2^
HD	Human density	Ordinal	Density of human living in the administrative area of the market as extracted by the online repository was categorized in three levels (low: 1–50, medium: 50–200, and high: >500 per km^2^)
IDEG	In-degree	Continuous	In-degree centrality of the market in the cattle trade network in Cameroon (ranging from 0 to 16 incoming trade connections per market or, in other words, of unique source markets of animals moving in each specific market)
MKT	Market	Categorical	The ID of the 31 livestock markets (M1 to M31) where the report data were obtained
WEEK	Week	Ordinal	The week of the year that the report was made as an ordinal variable (from the September 1, 2013, to the August 31, 2014)

The modeling was conducted using the *gamm4* package (31) in R statistical software version 3.2.3 (32). The mapping was generated using R statistical software (32) (version 3.2.3) using the *raster, rgdal*, and *ggplot2* packages and shp files obtained from the GADM database of Global Administrative Areas version 2.0 (www.gadm.org).

#### 2.3.2 Model Selection and Validation

The size of the dataset offered the opportunity to assess the variability of the model fit and to increase the confidence of its robustness and its predictive performance. As such, the dataset was split into two subsets: a testing set and a validation set (33). The former was used to model the data and select the final model, whereas the latter enabled us to evaluate the predictive performance of the model. For the purpose of this study, the validation set was composed of 10% of the entire data (*n* = 11,800) using a stratified random sampling method. This sampling method was used to ensure that a representative fraction for each commodity type was present.

Model selection was carried out using the information-theoretic approach (34). This approach is derived from ecological theory and consists of fitting various combinations of the putative drivers together for multiple sampled subset of the data to derive the final model from the set of possible candidates. In this study, 50 bootstrapped samples of equal size (*n* = 11,800) were randomly generated using a stratified approach from the testing dataset (i.e., resampling the data with replacement). For all 50 subsets, 24 candidate models were considered and compared to identify the final model (Table S2 in Supplementary Material). As recommended by Ref. (34), fitting performance was evaluated based on the Akaike Information Criterion (AIC (35)) and the adjusted coefficient of determination (*R*^2^) (33, 36). The AIC provides evidence for which combination of variables best explained the data with the minimal number of covariates, whereas the adjusted *R*^2^ statistic provides a more “global” measure of how good the model is at explaining the data by measuring the amount of variance explained by the model. It worth noting that both AIC and adjusted *R*^2^ are measures that penalize for the number of independent variables in the model. As such, these statistics provide measures of support for the most parsimonious model. For comparison, the ΔAIC was also extracted for each tested model by computing the difference between each model-specific AIC and the highest AIC among all tested models. The proportion of times each candidate model returned the lowest AIC value, *ϕ*, was also recorded. Calculation of this proportion determines the relative frequency that any candidate model is found to be the best (34).

For each candidate model, statistical significance for both linear and non-linear variables was set at *p* < 0.01. The proportion of times each variables were found significant in candidate models, *π*, was also computed. This proportion *π* provides a measure of support for each association, which due to the use of bootstrap resampling is robust to the effects of sampling error in the original data.

Predictive performance of the final models was evaluated by comparing model-based predictions with observed prices of cattle from the 11,800 transactions that were included in the validation dataset by using the adjusted *R*^2^ and the mean absolute error (MAE). The MAE measures the selection bias contributing to make the model inaccurate. By determining if the model has a positive or negative bias, it is possible to assess if the model is underestimating or overestimating the observed values (37).

## 3 Results

Over the entire dataset of transactions, live cattle were sold on average for 222,000 CFA (Q1–Q3 range: 170,000–290,000). Throughout the 52 weeks of the study period, the average price of cattle varied by ±4.5%, with a minimum during weeks of the dry season and a peak during the week before end-of-year festivities (Figure S3 in Supplementary Material). However, there was large variation in value between commodity types (i.e., bulls, steers, cows, young bulls, and heifers), with bulls and steers traded for the highest median price at 325,000 CFA (Q1–Q3 range: 291,000–390,000) and 338,000 CFA (Q1–Q3 range: 270,000–402,000), respectively (Table 2). In contrast, young stock (i.e., heifer and young bulls) were sold with a 23% discount in comparison with the average cattle price, at a median price of 170,000 CFA (Q1–Q3 range: 140,000–215,000).

**Table 2:** Descriptive statistics of live cattle prices recorded in markets of the Adawama and the West Regions in Cameroon.

	Bull	Cow	Steer	Heifer	Young bull	All categories
*n*	29,819	36,854	2,097	16,872	32,375	118,017
Overall	325 (291–390)	220 (193–250)	338 (270–402)	170 (140–201)	171 (140–215)	222 (170–290)
**Division**						
Vina	348 (307–400)	235 (200–270)	305 (260–374)	182 (150–220)	191 (155–230)	220 (175–290)
Djerem	306 (245–360)	200 (170–230)	320 (270–361)	146 (120–175)	162 (130–200)	185 (145–250)
Faro et Deo	301 (240–365)	169 (130–205)	302 (240–350)	132 (105–165)	128 (100–162)	150 (115–210)
Mayo Banyo	305 (271–338)	215 (192–248)	274 (218–320)	158 (142–182)	161 (147–175)	215 (179–285)
Mbere	342 (300–402)	208 (177–252)	363 (290–400)	162 (130–200)	181 (143–232)	220 (165–305)
Noun	338 (287–405)	232 (205–275)	396 (344–462)	180 (147–215)	175 (145–210)	250 (200–317)
**Season**						
Dry	328 (288–390)	220 (190–252)	344 (260–400)	172 (145–210)	175 (140–220)	226 (170–290)
Rainy	322 (300–390)	218 (200–245)	335 (275–400)	166 (142–214)	170 (140–200)	220 (160–290)

*n: total number of transactions reporting sale of live cattle*.

*All values are median (interquartile range) of the price (in ×1,000 CFA) at which individual live cattle have been sold in markets of the study area and stratified by administrative Divisions and season*.

*Noun is a Division of the West Region, whereas all others are Divisions of the Adamawa Region*.

**Figure 2:**
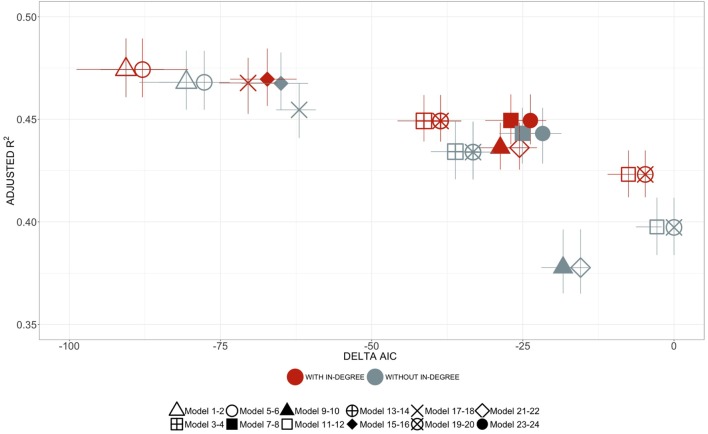
Effect of market centrality on models performance. The mean values of ΔAIC are displayed on the *x*-axis. The mean values of the adjusted *R*^2^ for each model, with the bars representing the ranges of variation across the 50 iterations, are displayed on the *y*-axis. The structure of each model is shown in Table S2 in Supplementary Material. The red color refers to models with in-degree centrality as a predictor, whereas gray color refers to models without in-degree. The shapes of the points refer to equivalent models, only differing by the inclusion or exclusion of in-degree centrality among the predictors. For example, the red square shape is equivalent to the gray square shape. The models are formally described in the Supplementary Material (Table S2 in Supplementary Material).

In total, 24 models were screened to identify factors affecting the price at which cattle were sold in Cameroonian markets. Each model included different *a priori* meaningful combinations of the nine predictor variables considered. Figure S2 and Table S2 in Supplementary Material provide the list of all models screened and the formal comparison of their fitting performance, respectively. Models 1 and 5 showed equivalent fitting performance with an adjusted *R*^2^ of 0.474 (range: 0.423–0.501) and 0.474 (range: 0.456–0.403), respectively, while minimizing their estimates of ΔAIC (Figure 2). Both models include: the week at which transactions were made; the age and sex of the animals involved in these transactions; the administrative division of the markets; the position (i.e., in-degree) of these markets in the cattle trade network in Cameroon; as well and the local densities of human and cattle reported at their location. Although Model 1 showed the lowest AIC in 82% of iterations, the additional factor included in this model, season, was not statistically significantly associated in any of the 50 iterations (*π* = 0%). Given that AIC and *R*^2^ measures from Models 1 and 5 were not significantly different (Figure 2), we considered Model 5 as the best, most parsimonious, model.

**Figure 3:**
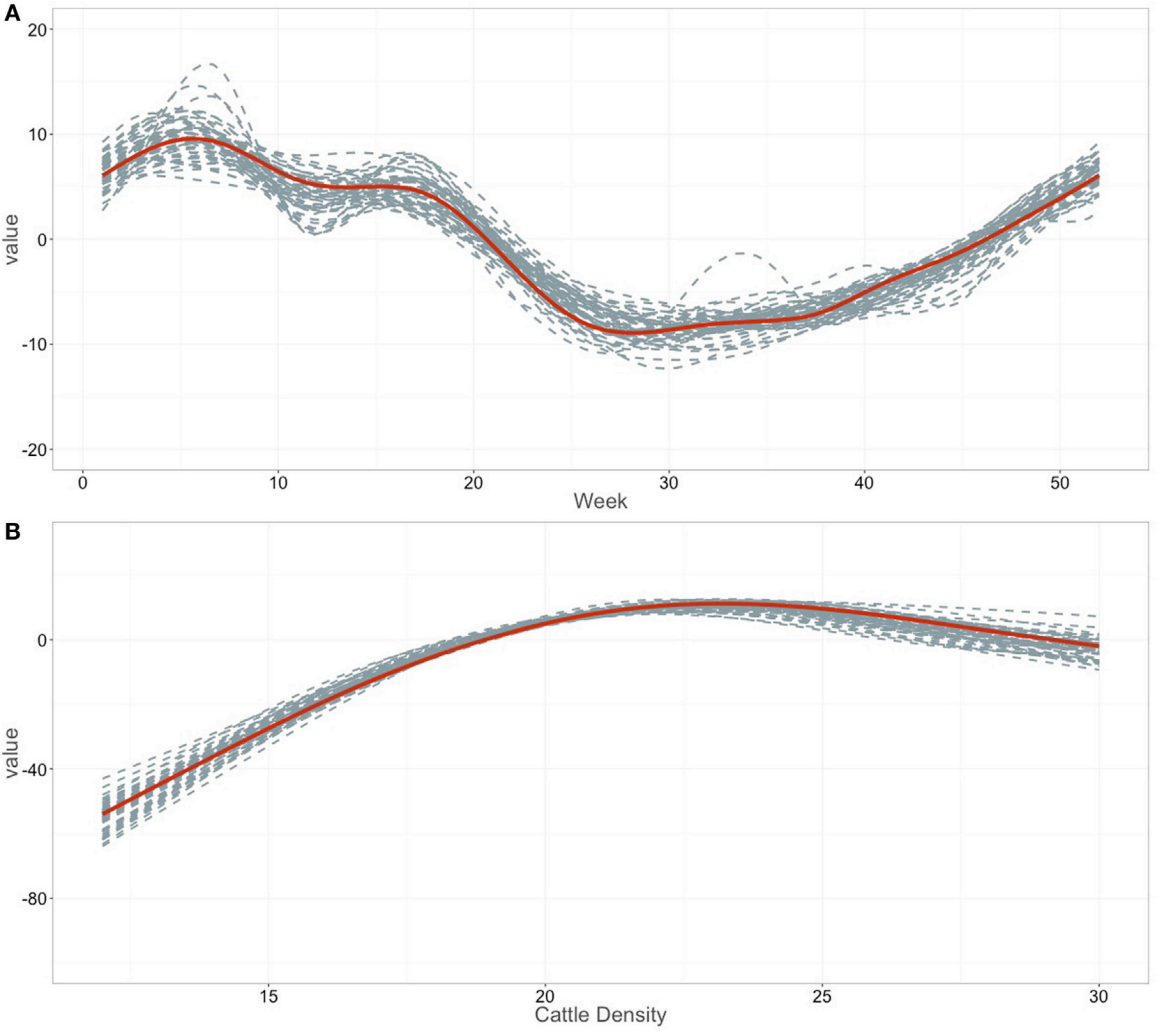
Smoothed fits of covariates. Smoothed fits of covariates modeling the relation with **(A)** the weekly observation and **(B)** the bovine population density. Tick marks on the *x*-axis are observed datapoints, and the *y*-axis represent the spline function. Gray dashed line indicates the smoothed fits for all the 50 iterations; the red solid line the mean smoothed fit across the 50 iterations.

Regression coefficients and their standard errors for all linear variables (i.e., the age and sex of the animals, the administrative division of the markets, in-degree, and human density) included in the final model are shown in Table 3, whereas the shapes of the functional forms for the non-linear variables (i.e., the week of the transaction, and bovine density) are shown in Figure 3. Although the temporal variations in the model is consistent with the empirical observations (Figure 3A), with the lowest price occurring during the dry season and a small peak prior the festive season, the week at which transactions were made had little influence on the mean price of live cattle (*π* = 0%). On the other hand, both animal age and sex were consistently associated with the value at point of trade, showing a *p* < 0.01 in all iterations of the model (Table 3). On average, individuals between 5 and 6 years of age consistently attained the highest price, amounting to 127,000 CFA more [95% confidence interval (CI): 123,668–130,332] than 0–2-year-old animals. However, cattle in Cameroon still retain significant value despite aging, with >10 year-old individuals worth on average 104,000 CFA more (95% CI: 98,296–109,704) than 0–2 year-old animals. Males on average attained a significantly higher price than females, with males (bulls and steers) 68,500 CFA (95% CI: 66,089–70,911) more expensive than females (cows and heifers).

**Table 3:** Mean estimates, and their associated variability and robustness, from the multivariable generalized additive mixed models of factors influencing the value (×1,000 CFA) of live cattle in markets of the Adawama and West Regions in Cameroon.

	Estimates (95% range)	SE (95% range)	*π*
**Intercept**	100.0 (91.4, 109.0)	7.70 (7.65, 7.74)	100%
**Age**			
0–2	Ref.	–	–
3–4	81.2 (78.1, 85.8)	1.58 (1.58, 1.58)	100%
5–6	127.0 (122.1, 130.4)	1.70 (1.69, 1.71)	100%
7–10	113.2 (108.0, 117.2)	1.97 (1.95, 1.99)	100%
>10	104.3 (98.6, 111.2)	2.91 (2.91, 2.92)	100%
**Sex**			
Female	Ref.	–	–
Male	68.5 (65.5, 71.4)	1.23 (1.22, 1.23)	100%
**Human density**			
Low	20.9 (14.2, 28.4)	7.41 (7.36, 7.45)	64%
Medium	Ref.	–	–
High	−6.59 (−17.2, 6.42)	12.3 (12.2, 12.3)	0%
**Network in-degree**	2.80^a^ (2.14, 3.66)	0.77 (0.76, 0.77)	96%
**Division**			
Vina	Ref.	–	–
Djerem	−34.5 (−40.9, −26.4)	6.82 (6.78, 6.86)	100%
Faro et Deo	4.00 (−3.18, 5.12)	10.34 (10.24, 10.48)	0%
Mayo Banyo	−31.5 (−36.1, −28.1)	6.20 (6.16, 6.24)	100%
Mbere	−11.6 (−17.1, −4.53)	7.69 (7.64, 7.74)	0%
Noun	−5.79 (−17.94, 9.52)	14.41 (14.22, 14.60)	0%
**Random effects**			
Market	2.4 (−8.4, 10,1)	11.24 (10.63, 11.81)	–

*Ref., reference; SE, standard error; *π*, proportion of significance defined as the number of iterations in which variables are significant (i.e., *p* < 0.01) over the 50 bootstrap iterations*.

*The variability of the regression coefficients was informed by the mean standard error (SE) whereas their robustness was informed by their 95% range computed over 50 bootstrap iterations. Confidence intervals (CI) for the linear variables can be calculated as the Estimate ± 1.96 × SE*.

*^a^ Interpretation: The price of cattle would, on average, increase by 2,800 CFA (95% C.I. 1,291–4,309) for each unique market sending animals to the specific market in which transaction occurred*.

The position of the market in the cattle trading system was consistently associated with price of traded cattle, showing a *p* < 0.01 in at least *π* = 96% of the iterations (Table 3). On average, the price of cattle would increase by 2,800 CFA (95% C.I.: 1,291–4,309) with each unit increase of in-degree centrality, corresponding to a unique source market. Although such an increase may seem minimal, in-degree was influential across all the models screened during the selection process, consistently showing better *R*^2^ and ΔAIC values than models where in-degree was dropped (Figure 2).

The trade price of cattle in markets was consistently associated with both the density of cattle (*π* = 96%) and the density of human populations (*π* = 64%) in the area where markets are located. Indeed, cattle prices were significantly higher in markets where the local human population density was low, with an additional 20,900 CFA (95% CI: 6,376–35,424) in the value of cattle compared to markets located in areas with medium human density (Table 3). At the same time, cattle were consistently 50,000 CFA cheaper in markets where cattle were at a low density (i.e., <13 per km^2^) and their value progressively increased with an increasing local cattle density until it reached a maximum price around 23 cattle per km^2^ (Figure 3B).

**Figure 4:**
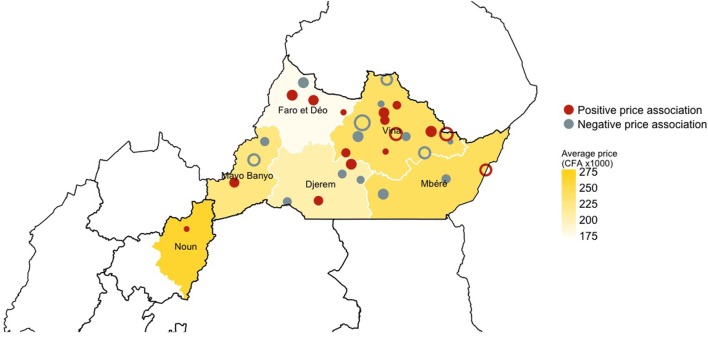
Geographical distribution of the markets and their estimated effect on the price of the traded cattle across the study area. Red color relates to markets with a positive association on price of live cattle, while gray color relates to markets which a negative association on price of live cattle. The size of the dots is proportional to the mean impact on price of the market over the 50 iterations: the bigger the dot the bigger the effect on the price. Open circles refer to the market that have a consistent, either positive or negative, effect on price across the 50 iterations. The intensity of the yellow background color, instead, relates to the mean price of animals traded in each division within the study area.

Despite adjusting for local conditions, a relevant variability in the price of cattle still remains across the study area. The price of cattle traded in the Djerem and Mayo Banyo Divisions of the Adamawa Region were consistently cheaper than the rest of the Divisions in our analysis (*π* = 100%, Table 3). On average, cattle traded in markets located in Djerem and Mayo Banyo Divisions were sold for 34,500 CFA (95% CI: 21,133–47,867) and 31,500 CFA (95% CI: 19,348–43,652) less than cattle sold in the Vina Division (Adawama), respectively. However, prices did not differ only between Divisions, live cattle were traded for significantly (*π* ≥ 96%) less than expected in four markets (Nyambaka, Likok, Mbe, and Banyo) whereas prices were inflated in three markets (Dibi, Djalingo, and Ngaoui) (Figure S4 in Supplementary Material). Figure 4 highlights the spatial distribution of market-level impacts (i.e., random effect) on the price at which cattle were sold once adjusted for all linear and non-linear predictors. Clearly, no particular spatial patterns were apparent. However, it is interesting to note that four of the seven markets for which prices of cattle deviate significantly from the adjusted average are located in the Vina Division (Adawama).

**Figure 5:**
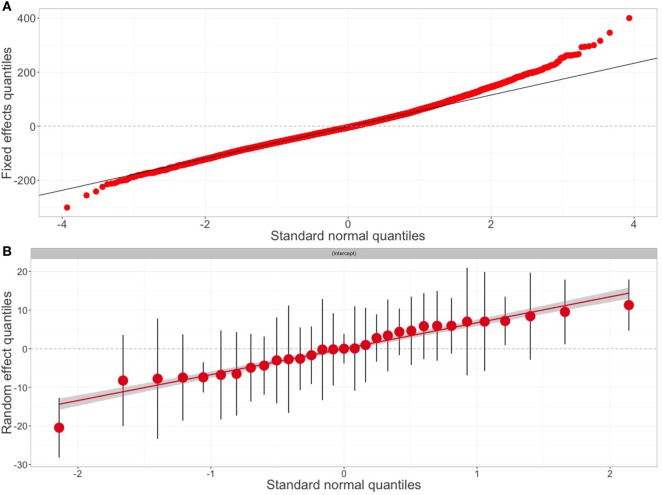
Goodness of fit of the final model. Scatter plots of the goodness of fit for the final model. Quantile–quantile plots of **(A)** the residuals errors and **(B)** random effects (31 markets) of the final model. The plots show a uniform distribution with only an increased deviation from normality for the extreme values.

Goodness-of-fit measures for the final model over the testing dataset are shown in Figure 2 and Table S2 in Supplementary Material. There was some variability in the value of cattle that was not explained in the model, with adjusted *R*^2^-values averaging around 0.474 (range: 0.456, 0.493). However, the quantile–quantile (QQ) plot of both the distribution of the residuals and random effects of the final model did not deviate massively from that expected under the null hypothesis (Figure 5). Acknowledging that imputed age data may have influenced the model outcomes, 50 iteration models were fitted over the reduced, non-imputed data. Whether the model used imputed aged data or not, little differences were found between estimates of the explanatory variables and between goodness-of-fit measures, confirming the robustness of the model outcomes.

**Figure 6:**
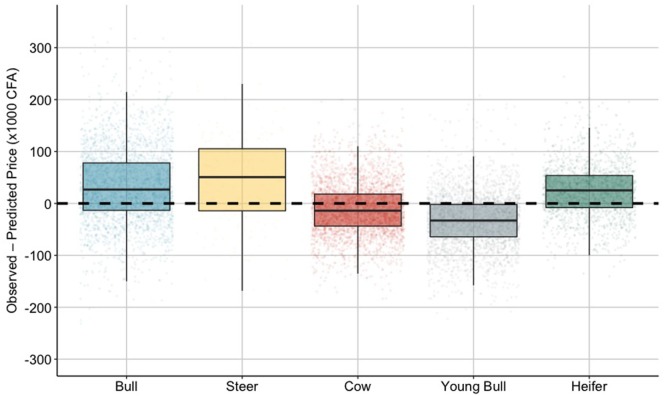
Predictive ability of the model. Boxplot of the difference between the observed and predicted prices (in CFA ×1,000) for each of the categories of traded cattle. For each box, dots represent the differential in prices for each records from the validation dataset. The upper, lower hinges and horizontal lines indicate the 1st and 3rd quartiles (the 25th and 75th percentiles) and the median of the distribution, respectively. Dashed horizontal line indicates perfect prediction.

We assessed the predictive performance of the model by examining how its predictions agree with the recorded cattle prices from the validation dataset. The adjusted *R*^2^ was 0.474 indicating that although a relatively high concordance exist between model inferences and recorded prices, only 47.4% of the variability in the data was accounted for. On average, predictions deviated from recorded prices by about 50,000 CFA (MAE = 49.6), equivalent to 23% of the mean cattle price across the study area. When comparing predictive ability of the model across the different cattle types, the price of bulls and steers was generally underestimated by a median of 26,800 CFA (Q1–Q3 range: −13,200–78,000) and 50,600 CFA (Q1–Q3 range: −14,200–105,200), respectively. Similarly, the price of heifers was underestimated by an average of 25,100 CFA (Q1–Q3 range: −8,000–53,600). In contrast, young bulls were traded, on average, for 32,900 CFA (Q1–Q3 range: 2,100–64,300) less than what we predicted based on our model (Figure 6).

## 4 Discussion

Many epidemiologist and policy makers have argued about the importance of local trading behavior on the efficiency and resilience of animal health management programmes implemented in rural communities. While incentives and compensations have been shown useful to increase the rate of reporting of infectious disease occurrence (38), these may be counter-effective if the compensation offered is too low or affect the sustainability of the programme if it is too high. Evaluating the prices at which livestock owners will be willing to report disease signs, while ensuring the sustainability of the programmes, is therefore critical. Yet, little information can be found in the literature on what affects the value of agricultural products (let alone for livestock commodities) in their local settings in SSA. Instead, research has been focused on the impact of shocks, either due to environmental changes, e.g., drought (18, 39), or fluctuations in global agricultural market prices (40, 41). These are important factors to consider when designing efficient animal health programmes. However, animal health programmes in SSA depend on long-term commitment of local rural communities that are heavily reliant on livestock production. In this context, governments face two key questions when setting prices: (i) what is the minimum price livestock owners would accept in compensation that provides sufficient motivation to report clinical signs and (ii) how variable are prices and how much local adjustment needs to be made in compensation. While the former would ensure the cooperation and willingness of livestock owners to report clinical signs, the latter would avoid undesirable effects on trade that may affect the ability of animal-health officers to predict (and control) diseases spread. Here, we focused on the second question, highlighting for the first time factors that determine the formation of live cattle price at livestock markets within the major pastoral and cattle production areas of central Cameroon.

As expected the value of animals varies between commodity types. Adult males (either bulls or steers) were the most expensive type of animal whereas young stocks (i.e., heifer or young bulls) were the cheapest (Table 2). Bulls and steers, are usually considered the most suitable for human consumption in various contexts (42, 43) and their value can be directly estimated by buyers. In contrast, females are mostly kept for breeding purposes in Cameroon, and their value is more long term, but also more uncertain as they may fail to breed in the future. However, healthy cows are still important assets in the herd and will only be traded for slaughter (usually at lower price) near the end of their reproduction life to recover money (12). It is therefore surprising that so many cows are being offered for sale in the study area. Lower prices were paid for young stock compared to adults, with a median 23% lower compared to the overall median price of live cattle. The low price of young bulls may be explained by either the large number of young bulls being offered for sale in the markets (27%, Table 2), or because of their lower weight or the uncertainty in their growth to optimal slaughter weight. On the other hand, heifers are typically kept in their herd of birth as replacements for unproductive cows. However, nearly 14% of the total number of animal traded in the study period were heifers. We can only speculate on the reasons why heifers were traded rather than kept in herds as replacement stock. Heifers could be perceived by herdsmen as a better animal to trade to generating rapid cash to pay bills or unforeseen expenses (4, 44). Alternatively, herdsmen may sell cattle (including heifers) if they suspect that they suffer from a health-related issue [i.e., as an emergency sale (45)]. As such, it is not surprising that the age and sex of the animals involved in transactions were found to be consistently associated with their price in the final predictive model (Table 3).

Cattle production in Cameroon depends almost exclusively on the traditional pastoralists who rely entirely on communal pasture land to meet the needs of their animals. Seasonal variations in the production and nutrient content of pastures have been correlated with the poor performance of cattle elsewhere in SSA (46), including Cameroon (47). Although our study only covers a single year of trading records, the period at which prices are minimal, visually coincides with the period during which pasture productivity is low (i.e., the dry season) resulting in poorer animal body conditions. However, temporal changes in cattle prices were minimal, representing less than 5% of the variation around the average cattle price. Also, we found poor evidence of effects of different periods of the year on live cattle price and no clear relation of higher prices during periods of religious festivities or national celebrations, as previously suggested elsewhere (15, 19). This may be because the study area is predominantly Muslim and sheep are more important in their religious celebrations. Although these results do not imply that external shocks, such as drought for instance, would not be influential in driving the price of cattle in the study area, these findings highlight that live cattle prices are robust to temporal factors and therefore these appear less important for setting compensation values.

The common wisdom in price formation is that supply and demand will regulate the price of commodities that are being traded (15). In the context of cattle prices, we would have expected prices to increase in markets located in areas where the cattle population density is low and the human population density is high. Conversely, markets in areas where the cattle population is high and the human population is low, should have recorded lower cattle prices. However, this relation was not linear in the present study. Our model shows that, on average, a 20,900 CFA and a 50,000 CFA premium were paid for cattle in markets located in low human population density and high cattle density, respectively. While these findings need to be regarded with caution, they should be considered and interpreted in relation to the specific features of the study area. The Adawama and West Regions represent the main cattle production areas in Cameroon, raising nearly a quarter of the national cattle herd (1), while also including a limited number of urban centers (23). It is clear that the large volume of cattle produced cannot be consumed by the local population and part of the production is redirected toward large urban centers outside the study area (such as Douala or Yaounde) or exported. While efforts were made to include markets from high consumption Regions outside the study area, records of market transactions in these Regions were not available and therefore inferences on price formation are only valid in the context of the study area.

Our previous assumption that population density was directly related to the consumption demand for live cattle and cattle meat fails to capture the structural complexity of the trading system in Cameroon. In particular, the network is not homogeneous and different actors operate at different levels in the network. Our results have shown that the average price paid for each animal is affected by the position of markets in the cattle trade network, with a premium of 2,800 CFA paid for each additional unique market from which they source animals (in-degree). Although the reasons why animals would be purchased for greater price in more central markets are not totally clear, it is believed that this might be related to greater demand for live animals for either slaughter or re-sell. At the periphery of the network are large numbers of livestock owners selling into the market to relatively few buyers and very few butchers/dealers. As animals move through the network to more central markets (i.e., with higher in-degree), the profile of actors changes with local dealers selling batches of cattle to a large population of butchers and traders who are trading directly to large urban centers outside the study area.

Previously it has been shown that increasing the number of incoming connections to nodes of a network, in our case markets, could promote the circulation of pathogens, particularly rapidly spreading infectious diseases (48–50). As such, targeting key markets as part of surveillance strategies has the potential to increase the disease detection sensitivity of the surveillance system. Several studies have been carried out in SSA to investigate the structure and dynamics of livestock trade networks (14, 49, 51). However, these networks are difficult to compile, usually through carrying out questionnaire interviews to traders and livestock owners and may suffer multiple methodological limitations that may bias inferences. In this study, we have established for the first time the link between market position in the trade network with the price at which animals were sold. If such a link is confirmed, it would provide an additional tool to policy makers for identifying highly connected markets upon which surveillance and control activities may be implemented.

In the current study, the size of the available dataset allowed us to apply an iterative modeling approach to assess the variability of the model and increase the confidence in the robustness and validity of the relationship between the price of cattle and the putative drivers of price formation (52). However, the price of cattle across the study area was not totally explained by our model, with 52.6% (range: 50.7–54.4%) of the variations still unaccounted for. In particular, our model tends to overestimate the price of young bulls but underestimates the price of adult males. Both phenotypic and breed characteristics of the animals involved in transactions, when adjusted with local and temporal factors, have the potential to explain a large proportion (>60%) of variations in their selling price (53, 54). For example, cattle that appear lighter, sick or having physical impairments are likely to be sold at a discounted price, whereas animals with large humps (which is a delicacy in Cameroon), or particular breeds such as the Gudali (1), would be sold at a large premium. Alternatively, requiring cattle to travel long distances (either on foot or using a vehicle) to be sold at market has been associated with larger livestock prices in SSA (21), accounting for up to 70% of the transaction costs (19). Although including these animal-level details (e.g., transport, breed, body condition) in the analysis would have been a huge refinement to our understanding of price variations among cattle sold in Cameroon and would have allowed comparisons with other studies, these data were not available in the records from the markets involved in our study.

In this study, we assumed that breed distributions in Cameroon are related to administrative Divisions. As such, we expected that the geographical drivers (captured as the Divisions in the model) would account for the influence of breed and cultural factors on cattle prices. Interestingly, most of the markets showing significant deviation from the average price (i.e., random effects) were in the Vina Division (Figure 4) where the Gudali breed predominates and where access to transport infrastructure such as rail and more recently road is available. It is therefore likely that the variable Division would act a proxy and account for the influence of both breed and transport onto cattle prices. However, such information need to be ultimately recorded if we want to better understand the animal health status of cattle at markets, as well as better understand how cattle prices are formed.

Limitations in the availability of local and regional data on the price of cattle sold in markets also limited our ability to assess whether price in other regions of the country, or in other neighboring countries, were influencing the price of cattle sold in the study area (40). As such, it was not possible to assess the level of protection of the local trading system against external market shocks (41) and, thus, evaluate the level of market integration of the cattle trading system in the study area. Consequently, we were not able to evaluate the level of vulnerability of stakeholders involved in the local and regional cattle industry (in broad term) against external factors. Again, improving data collection procedures at livestock markets is of the utmost importance if we want to develop integrated animal health management programmes. However, rather than being restricted to our study area, prices at which cattle are sold need to be consistently and regularly recorded (as well as centrally kept) in the wider SSA to facilitate such a study.

In conclusion, we have shown that cattle prices in Cameroon vary between commodity types, geographical areas, and the position of the market in the national trading network. However, there remains a large unexplained component in the price formation that may be due to breed, body condition, culture, and access to transport that are not currently captured in the trade records from markets. In addition, this study was not able to include markets in other administrative Regions of Cameroon and in neighboring countries that may also have an impact on price, particularly when including market in urban centers in high consumption areas. Nevertheless, this study represents a milestone in better understanding the cattle trading system and price formation in a Central African country and provides valuable information for better design of animal health programmes and for epidemiologists to develop better dynamic mathematical models for exploring disease spread and the impact of alternative control measures.

## Ethics Statement

This research was authorized by the Ministry of Livestock, Fisheries and Animal Industries (MINEPIA) and approved by the Cameroon Academy of Sciences. In the United Kingdom, approval was given by the Veterinary Ethical Review Committee of the Royal (Dick) Veterinary School (University of Edinburgh). All methods for data collection and gathering were performed in accordance with the relevant regulations and in compliance with the received guidelines.

## Author Contributions

PM, IH, VT, KM, and BB designed the data collection and the overall research programme; PM, VN, and SH performed the field work. PM, TP, IH, and BB designed the study. PM conducted the analyses. PM, GR, and TP interpreted the results and wrote the manuscript. GR, IH, KM. and BB revised and reviewed the manuscript.

## Conflict of Interest Statement

The authors declare that the research was conducted in the absence of any commercial or financial relationships that could be construed as a potential conflict of interest.
