# Supplementary material for: Drivers of Live Cattle Price in the Livestock Trading System of Central Cameroon
Source: Front Vet Sci. 2018 Jan 17;4:244. doi: 10.3389/fvets.2017.00244 (PMC5776083; doi:10.3389/fvets.2017.00244)
Supplement: Supplementary file 1 [file image_1.PDF]

Supplementary Material: Drivers of live cattle price in the livestock trading system of Central Cameroon

Paolo Motta *, Ian G. Handel, Gustaf Rydevik, Saidou M. Hamman, Victor Ngu Ngwa, Vincent N. Tanya, Kenton L. Morgan, B. Mark de C. Bronsvoort and Thibaud Porphyre

*Correspondence:
Paolo Motta
Paolo.Motta@roslin.ed.ac.uk

1 SUPPLEMENTARY TABLES

Table S1. Correlation coefficients between nodes centrality measures.

	**Betweenness**	**Degree**	**In-Degree**	**Out-Degree**	**Eigenvector**
**Betweenness**	1	0.8078	0.7324	0.7198	0.7087
**Degree**	0.8078	1	0.8032	0.7642	0.8879
**In-Degree**	0.7324	0.8032	1	0.7455	0.7731
**Out-Degree**	0.7198	0.7642	0.7455	1	0.7911
**Eigenvector**	0.7087	0.8879	0.7731	0.7911	1

Table S2. Variables and goodness-of-fit measures, and their range, for all models considered.

**Model**	x_k	z_m	R^2	**ΔAIC**	ϕ
**1**	HD+IDEG+SEAS+DIV	CD+T	47.4 (42.3, 50.1)	-91.1 (-103, -81.2)	86%
**2**	HD+SEAS+DIV	CD+T	46.8 (45.2, 48.2)	-81.1 (-89.5, -74.8)	0%
**3**	HD+IDEG+SEAS	CD+T	44.9 (43.9, 46.8)	-41.7 (-46.2, -37.3)	0%
**4**	HD+SEAS	CD+T	43.4 (43.9, 46.8)	-36.2 (-41.2, -31.9)	0%
**5**	HD+IDEG+DIV	CD+T	47.4 (45.6, 49.3)	-88.0 (-99.4, -78.8)	14%
**6**	HD+DIV	CD+T	46.8 (45.2, 48.6)	-77.9 (-86.1, -72.1)	0%
**7**	IDEG+SEAS+DIV	CD+T	45.0 (43.4, 46.8)	-26.8 (-31.7, -22.6)	0%
**8**	SEAS+DIV	CD+T	44.2 (42.6, 46.1)	-24.9 (-30.4, -16.6)	0%
**9**	HD+IDEG+SEAS+DIV	T	43.6 (42.6, 45.6)	-28.5 (-33.6, -24.4)	0%
**10**	HD+SEAS+DIV	T	37.8 (36.2, 40.1)	-18.6 (-22.0, -18.6)	0%
**11**	IDEG+SEAS	T	42.3 (41.0, 44.2)	-7.9 (-11.5, -5.9)	0%
**12**	SEAS	T	39.7 (38.2, 41.6)	-3.1 (-6.9, -1.9)	0%
**13**	HD+IDEG	CD+T	44.9 (43.3, 46.7)	-38.6 (-42.3, -34.5)	0%
**14**	HD	CD+T	43.4 (42.7, 45.2)	-33.0 (-36.3, -29.1)	0%
**15**	IDEG+DIV	CD+T	46.2 (44.3, 48.6)	-67.8 (-76.3, -61.8)	0%
**16**	DIV	CD+T	46.6 (45.1, 48.6)	-65.3 (-70.2, -60.0)	0%
**17**	HD+IDEG+DIV	T	46.1 (44.8, 48.4)	-70.3 (-75.6, -65.4)	0%
**18**	HD+DIV	T	45.3 (43.9, 47.2)	-62.1 (-66.0, -59.1)	0%
**19**	HD	CD	42.3 (41.0, 44.2)	-4.7 (-6.5, -3.2)	0%
**20**	IDEG	CD+T	39.6 (38.2, 41.6)	0.0 (0.0, 0.0)	0%
**21**	HD+IDEG	T	43.6 (42.2, 45.7)	-25.3 (-28.3, -21.5)	0%
**22**	HD	T	37.8 (36.3, 40.0)	-15.4 (-17.4, -14.0)	0%
**23**	IDEG	T	44.9 (43.3, 46.8)	-23.7 (-26.9, -19.8)	0%
**24**	–	T	44.2 (42.6, 46.0)	-21.7 (-25.4, -16.8)	0%

x_k: Linear predictor variables; z_m: non-linear predictor variables; R^2: Adjusted coefficient of determination between observed and predicted data; ΔAIC: Difference of Akaike information criterion between models; ϕ: Number of iterations in which candidate model returned the lowest AIC value over the 50 bootstrapped iterations.

Linear predictor variables: HD: Human density; IDEG: Market-level centrality measure as defined by in-degree; SEAS: Season at which transaction occurred; DIV: Administrative division of the market.

Non-linear predictor variables: CD: Cattle density of the market; T: Week at which transaction occurred.

For all models, age and sex of the animal involved in each recorded transaction were included as linear predictor variables, whereas market at which transaction occured was included as random effect.

2 SUPPLEMENTARY FIGURES

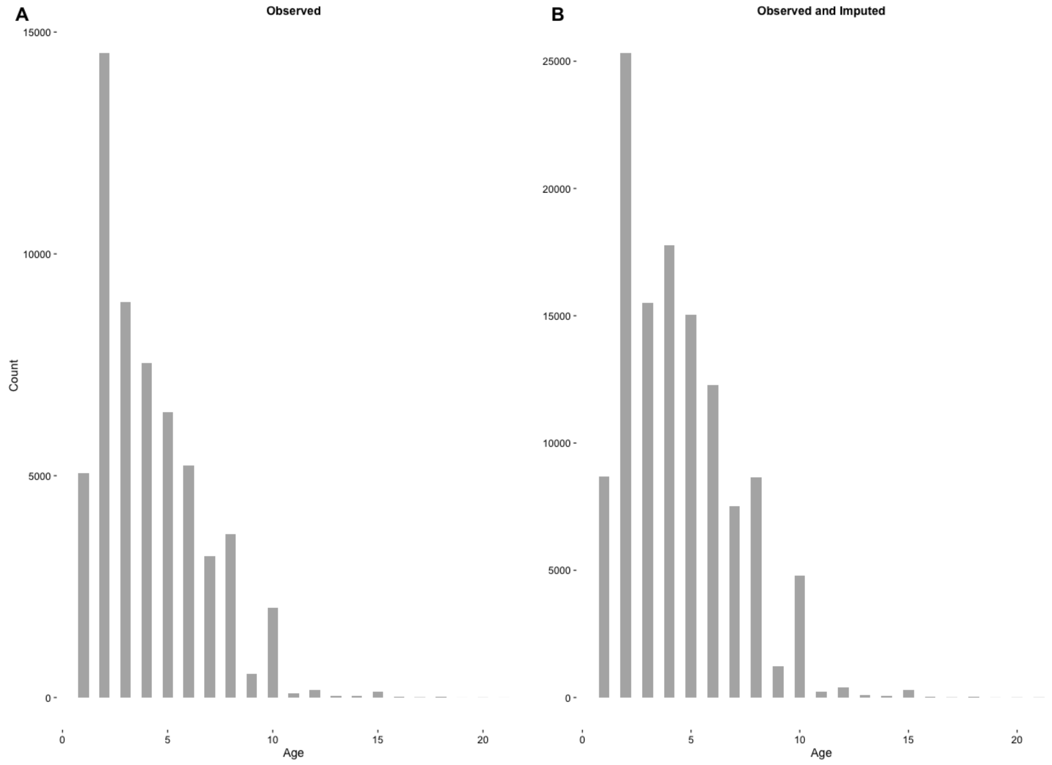

Figure S1. Age distribution of traded cattle.
A: age distribution of the incomplete dataset. B: age distribution after imputation of missing age in the dataset. The y axis ranges in A and B are in different scales as the intent of the figure is to control for the pattern of distribution of the data points.

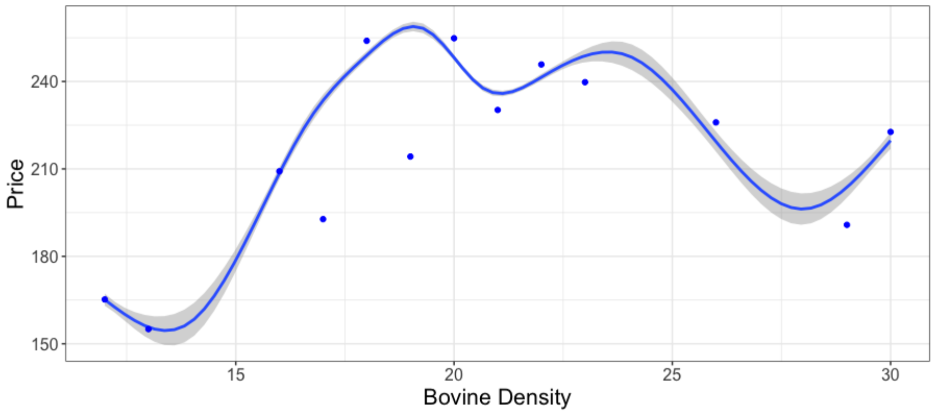

Figure S2. Relation between cattle price and cattle population density.
On the y axis the price of the traded cattle and on the x axis the cattle population density in heads per squared kilometre. The blue line represents the smoothed line of the relation between the price of the 118,017 traded cattle and the estimated cattle population density in the local administrative unit for the year 2005 as obtained from the online FAO GeoNetwork repository.

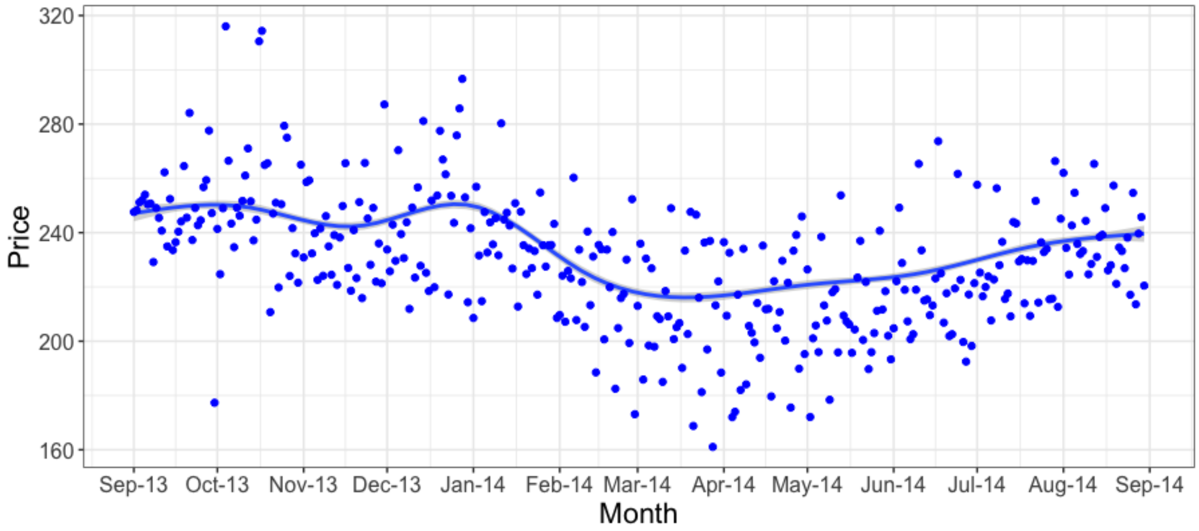

Figure S3. Mean price over 52 weeks from the 1st September 2013 to the 31st August 2014. Mean price per animal (CFA x 1,000): the blue dots represent the mean price per animal in that week, the blue line the trend over the 52 weeks time and the grey shade the smoothing terms of the linear relation between the two variables.

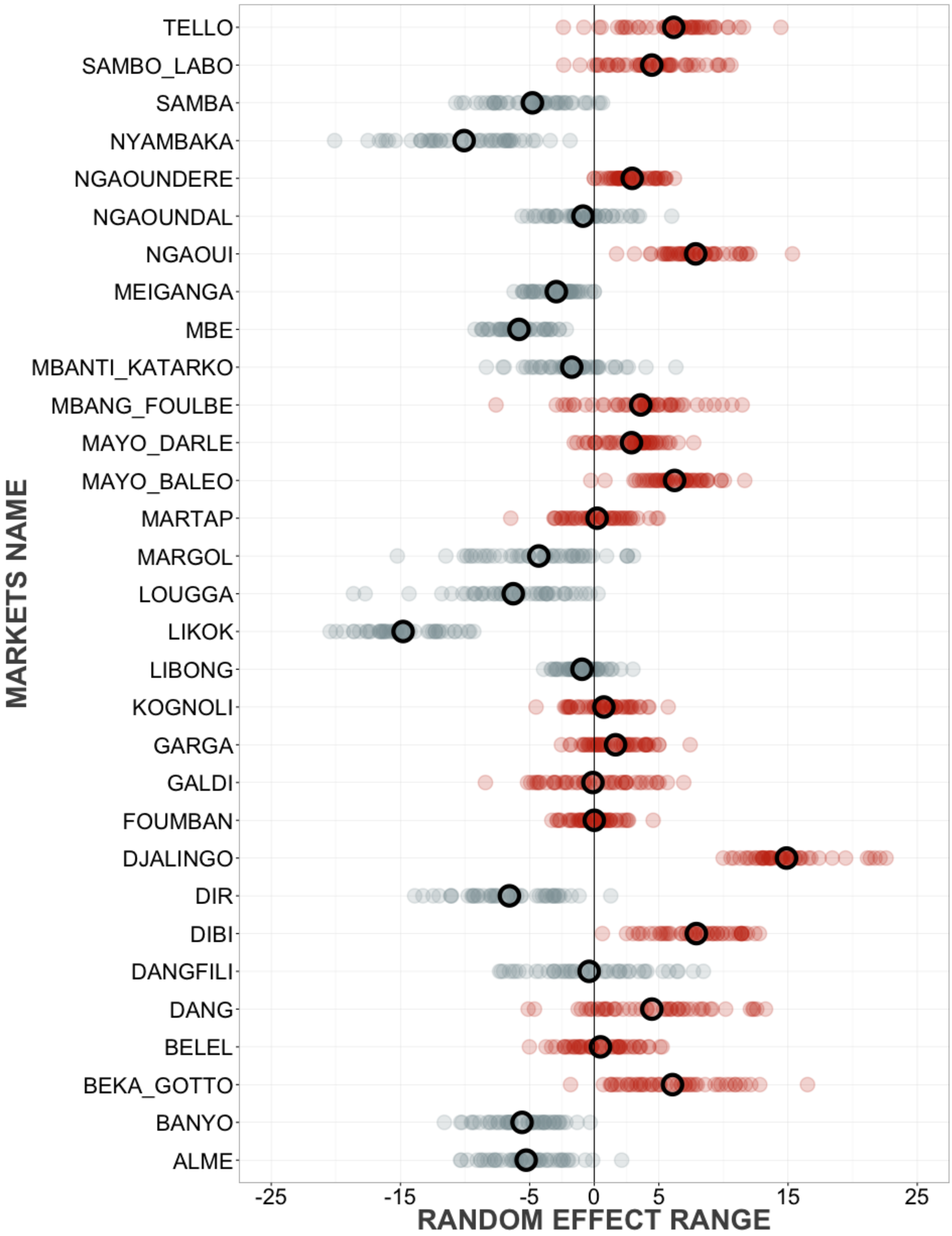

Figure S4. Plot of the *random effect* estimates for each market.
Inferred values for each of the 31 markets included in the study. Red dots relate to markets with a mean positive association on price of live cattle, while grey dots relate to markets with a mean negative association on mean price of live cattle. The black circle indicates the mean output over the 50 iterations.
